# DCS blood flow index underestimates skeletal muscle perfusion *in vivo*: rationale and early evidence for the NIRS-DCS perfusion index

**DOI:** 10.1117/1.JBO.29.2.020501

**Published:** 2024-02-06

**Authors:** Miles F. Bartlett, Andrew P. Oneglia, Mark D. Ricard, Ashfaq Siddiqui, Erin K. Englund, Erin M. Buckley, Dennis M. Hueber, Michael D. Nelson

**Affiliations:** a University of Texas at Arlington, Arlington, Texas, United States; b Bartlett Sciences LLC, Dallas, Texas, United States; c Total Vascular Wellness, Mansfield, Texas, United States; d University of Colorado Anschutz Medical Campus, Aurora, Colorado, United States; e Georgia Institute of Technology and Emory University, Atlanta, Georgia, United States; f ISS Inc., Champaign, Illinois, United States

**Keywords:** diffuse correlation spectroscopy, blood flow index, perfusion, exercise, near-infrared spectroscopy

## Abstract

**Significance:**

Diffuse correlation spectroscopy (DCS) permits non-invasive assessment of skeletal muscle blood flow but may misestimate changes in muscle perfusion.

**Aim:**

We aimed to highlight recent evidence that DCS blood flow index (BFI) misestimates changes in muscle blood flow during physiological perturbation and to introduce a novel approach that adjusts BFI for estimated changes in vasodilation.

**Approach:**

We measured changes in muscle BFI during quadriceps and forearm exercises using DCS, the latter of which were adjusted for estimated changes in microvascular flow area and then compared to Doppler ultrasound in the brachial artery. Then, we compared adjusted BFI- and arterial spin labeling (ASL) MRI measures of gastrocnemius blood flow during reactive hyperemia and plantar flexion exercise.

**Results:**

We observed little-to-no change in quadriceps BFI during maximal-effort exercise. Similarly, forearm BFI was modestly increased during handgrip exercise, but the magnitude was significantly lower than measured by Doppler ultrasound in the brachial artery. However, this difference was ameliorated after adjusting BFI for estimated changes in microvascular flow area. Similar observations were also observed in the gastrocnemius when directly comparing the adjusted BFI values to ASL-MRI.

**Conclusions:**

Adjusting BFI for estimated changes in microvascular flow area may improve DCS estimates of muscle blood flow, but further study is needed to validate these methods moving forward.

## 1 Introduction

Diffuse correlation spectroscopy (DCS) is a non-invasive, optical technique that has been used to relate rapid fluctuations in light intensity of a single/few detected speckles to the motion of red blood cells (RBCs) in the underlying tissue.^1^^,^^2^ The mathematical units for DCS estimates of tissue perfusion (typically called a “blood flow index” or BFI) are ${\mathrm{cm}}^{2}\cdot s^{-1}$, although it is more common to report changes in tissue blood flow as a function of time, i.e., %- or fold-change from a resting baseline, by normalizing BFI to a resting baseline period.^3^^,^^4^ Importantly, while changes in DCS BFI are thought to be driven by changes in RBC velocity, *in silico* studies by Boas et al. demonstrated that changes in hematocrit or blood vessel diameter can also affect the relative change in DCS BFI,^5^ which Sathialingam et al. recently corroborated *in vitro* using a two-dimensional (2D), single-plane phantom model.^6^ Moreover, Boas et al. also found that DCS BFI can be affected by shear-diffusion, which describes differences in RBC velocity at different points within a microcapillary (i.e., faster RBC velocity in the lumen isocenter and slower velocities near the capillary walls).

As such, changes in DCS BFI during physiological perturbation will match changes in tissue perfusion (mL/100  g tissue/min) only if there are no changes in: (1) blood vessel diameter, (2) shear-diffusion dynamics, and (3) hematocrit. However, the recent 2D phantom work by Sathialingam et al. showed that photons can indeed pass through blood vessel diameters as large as 85 to 100  μm, a size that would certainly be susceptible to vasodilation in human muscle *in vivo*.^6^ This suggests that the assumptions of constant blood vessel diameter, shear-diffusion dynamics, and microvessel hematocrit may not be physiologically valid *in vivo*. Indeed, the relative change in DCS BFI in muscle is consistently lower than the relative change in either Doppler-derived conduit artery blood flow^3^ or microvascular perfusion measured by arterial spin labeling (ASL) MRI.^7^

Here, we report several recent observations that highlight an uncoupling between DCS BFI from changes in skeletal muscle perfusion *in vivo*. We hypothesize that DCS BFI in skeletal muscle is confounded by changes in vasodilation/constriction that alter the microvascular flow area (${\mathrm{MV}}_{A}$), which would allow net tissue perfusion to increase or decrease independent of changes in RBC velocity. Subsequently, we provide preliminary evidence for a mathematical adjustment that appears to overcome these confounding limitations *in vivo*, by adjusting BFI for changes in frequency domain near-infrared spectroscopy (NIRS)-derived total-heme content (HbTot) normalized to the magnitude of the NIRS-derived Hb-nadir.

## 2 Methods

All study procedures were approved by the University of Texas at Arlington and University of Texas Southwestern Medical Center Institutional Review Boards. All subjects gave written informed consent to participate prior to being enrolled.

### 2.1 Experimental Procedures

Part-1: Single-leg knee extension: nine healthy young subjects (four female; ½ skinfold thickness 6.8±2.3  mm) were seated upright with their right ankle strapped to a Biodex-3 dynamometer (Biodex Medical Systems) and a NIRS-DCS sensor secured over the vastus lateralis (VL). The protocol consisted of a 2 min resting baseline, 4 min of maximal-effort isokinetic knee extensions (one contraction every 2 s, $120{}^{\circ}\cdot s^{-1}$), and ∼10  min of passive recovery. To avoid contraction-induced motion artifact in the BFI signal, subjects skipped a contraction at 24, 60, and every 30 s thereafter so that BFI could be measured while the muscle was motionless.

Part-2: cycling exercise: four healthy young subjects (two F; ½ skinfold thickness 6.4±1.8  mm) performed an incremental cycling test to volitional fatigue on an electronically braked cycle ergometer (Corival, Lode B.V.). Following 2 min of resting baseline, the initial workload was set to 30 W and increased by 30 W every 3 min until volitional fatigue, followed by ∼12  min of passive recovery. Consistent with part-1, the NIRS-DCS sensor was positioned over the VL. Subjects briefly stopped cycling at the end of each stage so that BFI could be measured while the muscle was motionless.

Part-3: Handgrip exercise: the experimental procedures for part-3 have previously been described in detail.^8^ Briefly, following 2 min of resting baseline, 12 healthy young subjects (1 F; ½ skinfold thickness 5.9±2.3  mm) performed 5 min of moderate-intensity handgrip exercise and 5 min of passive recovery. The NIRS-DCS sensor was positioned over the flexor digitorum profundus. Doppler ultrasound of the brachial artery was used to assess changes in conduit blood flow. Changes in BFI were measured between contractions when the muscle was motionless.

Part-4: ASL MRI: using a previously described MRI sequence,^9^ medial gastrocnemius muscle perfusion was measured by ASL-MRI in three healthy young males (supermuscular tissue thickness 3.4±0.9  mm) during reactive hyperemia and after plantar flexion exercise. An MRI-compatible NIRS-DCS sensor was positioned over the medial gastrocnemius for concurrent acquisition of tissue BFI and oxygenation. The reactive hyperemia protocol began with a 2.5 min resting baseline, followed by 8 min of arterial cuff occlusion, and 10 min of recovery. The participants then performed plantar flexion exercise using a custom-built MRI compatible ergometer, with perfusion measured throughout 2 min of baseline and 10 min of recovery.

### 2.2 Experimental Instruments

All NIRS-DCS data were continuously collected at 5 Hz using a commercially available device (MetaOx, ISS Inc.) that consists of a four channel frequency domain NIRS system operating at six wavelengths (690, 700, 730, 750, 785, and 830 nm) coupled with a four channel DCS system operating at 850 nm.^8^^,^^10^ The optical sensor consisted of a single 2.4 cm source–detector pair for DCS and four pairs for FDNIRS (2.0, 2.5, 3.0, and 3.7 cm). For FDNIRS, measured AC amplitude attenuation and phase shift as a function of source–detector separation were fit to the semi-infinite solution of the photon diffusion equation to estimate the wavelength-dependent absorption ($\mu_{a}$) and reduced scattering ($\mu_{s}^{\prime }$) coefficients. Oxy- and deoxy-hemoglobin (${\mathrm{HbO}}_{2}$ and HHb, respectively) were derived from measurements of $\mu_{a}$ using the known hemoglobin extinction coefficients; total hemoglobin (HbTot) was defined as ${\mathrm{HbO}}_{2}+\mathrm{HHb}$. For DCS estimates of BFI, the measured normalized intensity autocorrelation functions were fit to the correlation diffusion equation, assuming a semi-infinite medium. To account for exercise-induced changes in tissue optical properties, a 3-s rolling average of the $\mu_{a}$ and $\mu_{s}^{\prime }$ values extrapolated to 850 nm were incorporated into the calculation of BFI at each timepoint.

For part-3, brachial artery blood flow (BABF) (mL/min) was measured using duplex Doppler ultrasound (Vivid-I, GE Healthcare), as described previously.^8^ A Doppler audio transformer was used to convert the Doppler audio signal into real-time, continuous measurements of blood velocity.^11^ Brachial diameter was measured using B-mode ultrasound imaging and commercially available image recording software (Vascular Imager, Medical Imaging Applications LLC, Coralville, Iowa) and then postprocessed using FMD Studio Cardiovascular Suite software (QUIPU, Pisa, Italy).

### 2.3 Adjusting DCS BFI for Estimated Changes in Vasodilation

Flow through blood vessels can be measured from the volumetric flow rate equation:

Q=vA, (1)

where Q is the flow rate, v is the mean blood velocity, and A is the area of the blood vessel; the latter of which is calculated as $\pi r^{2}$ (assuming a cylindrically shaped vessel). Because DCS BFI is largely a measure of RBC velocity ($v_{\mathrm{RBC}}$), it only accounts for the v component of Eq. (1). To overcome this limitation, we propose that changes in muscle blood flow measured by DCS BFI can be improved by estimating changes in microvessel area from changes in total tissue heme-content (HbTot). First, we assume that if hematocrit and RBC density remain relatively constant, then changes in HbTot can only be achieved by changes in microvessel area (${\mathrm{MV}}_{A}$). To normalize changes in HbTot to “baseline,” such that the units for change are consistent with those used for DCS BFI during physiological perturbation (i.e., fold-change from baseline), changes in HbTot are normalized to the magnitude of the NIRS-derived Hb-nadir; measured as the difference between peak deoxyhemoglobin (HHb) during prolonged ischemia and the minimum HHb observed during recovery.^12^ Mathematically, this takes the form of

$$\Delta {\mathrm{MV}}_{A}(t)=(\frac{\;((\mathrm{HbTot}(t)-\mathrm{Hb}\text{ Totbaseline})+\mathrm{Hb}-\mathrm{Nadir})}{\mathrm{Hb}-\text{Nadir}}),$$ (2)

where $\Delta {\mathrm{MV}}_{A}$ is the scaling factor for estimating the fold-change in microvessel area at timepoint t; HbTot(t) and HbTotbaseline are the HbTot (in μmol) measured at timepoint t and baseline, respectively; and Hb-nadir is the magnitude of change in deoxyhemoglobin (in μmol) observed during a prolonged (5+ min) arterial occluson.^12^ Importantly, the heme-nadir represents the fraction of the NIRS signal that emanates from the gas-conducting microvasculature, which should, theoretically, also represent the microvessels that influence DCS BFI. Equation (1) for DCS BFI therefore becomes

$$\mathrm{BFI}\text{-}{\mathrm{Adj}}_{\mathrm{LIN}}(t)=\Delta v_{\mathrm{RBC}(t)}*\Delta {\mathrm{MV}}_{A(t)},$$ (3)

where $\mathrm{BFI}\text{-}{\mathrm{Adj}}_{\mathrm{LIN}}$ is the adjusted BFI value, $\Delta v_{\mathrm{RBC}}$ is the fold-change in RBC velocity measured via DCS BFI at timepoint t (i.e., how BFI is typically calculated and reported in muscle; ${\mathrm{BFI}}_{t}/{\mathrm{BFI}}_{\text{baseline}}$), and $\Delta {\mathrm{MV}}_{At}$ is the fold-change in microvessel area at timepoint t, as calculated in Eq. (2). Importantly, $\mathrm{BFI}\text{-}{\mathrm{Adj}}_{\mathrm{LIN}}$ assumes that $\Delta {\mathrm{MV}}_{A}$ is linearly related to changes in HbTot. Alternatively, if we use assume that $\Delta {\mathrm{MV}}_{A(t)}$ reflects a change in microvessel radius, and the area of a circle is given by $\pi r^{2}$, then we can also adjust DCS BFI as

$$\mathrm{BFI}\text{-}{\mathrm{Adj}}_{\pi r2}(t)=\Delta v_{\mathrm{RBC}t}*({\Delta \mathrm{MV}}_{A(t)}{)}^{2},$$ (4)

where $\Delta v_{\mathrm{RBC}}$ and $\Delta {\mathrm{MV}}_{A}$ have the same definitions as in Eqs. (2) and (3), and $\mathrm{BFI}\text{-}{\mathrm{Adj}}_{\pi r2}$ is the adjusted DCS BFI value when $\Delta {\mathrm{MV}}_{A}$ is squared. Importantly, when quantifying changes in BFI as a fold-change above baseline, then the π-values from timepoint (t) and baseline cancel each other out, leaving $\Delta r^{2}$ (i.e., $\Delta {\mathrm{MV}}_{A}^{2}$) as the only variable driving the adjustment.

### 2.4 Statistical Analyses

All statistical analyses were conducted in SPSS v29.0 (IBM Inc.) using univariate ANOVAs. All group data are reported as mean ± SD.

## 3 Results

**Fig. 1:**
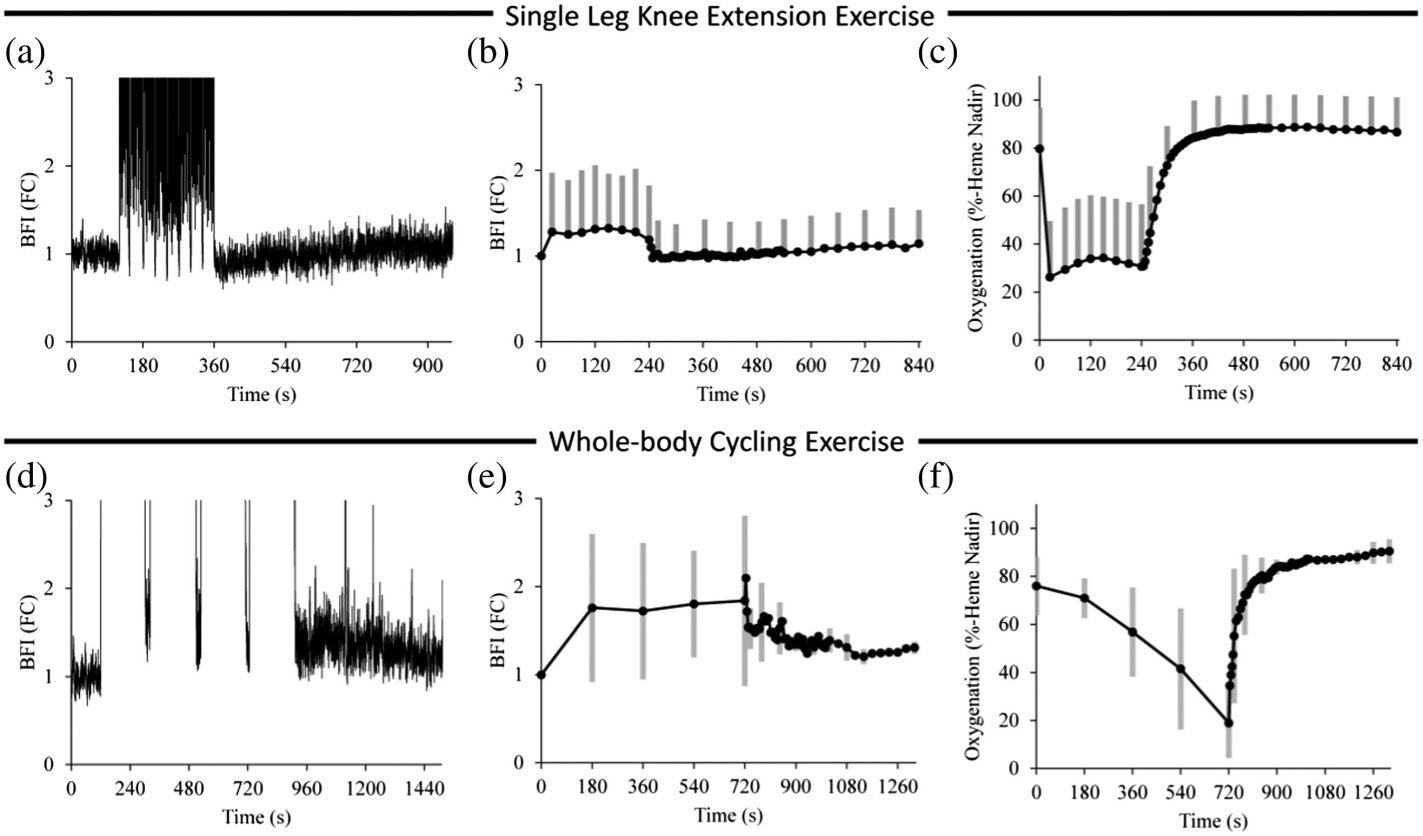
DCS BFI and muscle oxygenation during maximal-effort knee-extension exercise (top) and whole-body cycling exercise (bottom). (a), (c) Changes in DCS BFI (fold-change above baseline; FC) from a representative subject recorded throughout 2 min of resting baseline, high-intensity exercise, and 10 min of passive recovery. (b), (e) Group changes in DCS BFI (fold-changes for both) during exercise and throughout recovery. (c), (f) The decreases in muscle oxygenation that were observed during exercise. Note that in both cases, DCS showed little-no change above baseline despite substantial decreases in muscle oxygenation, indicating that the muscle mass beneath the DCS probe was indeed active. Group data are presented as mean ± SD.

As shown in Fig. 1, we observed only minor increases in quadriceps BFI above baseline following high-intensity knee extension (top) and cycling (bottom) exercises, despite marked decreases in muscle oxygenation. This finding is inconsistent with numerous investigations measuring femoral artery blood flow during knee extension^13^ or cycling exercises^14^ and suggests that increases in quadriceps perfusion are primarily driven by increases in vasodilation, thereby uncoupling the relationship between muscle perfusion and DCS BFI.

**Fig. 2:**
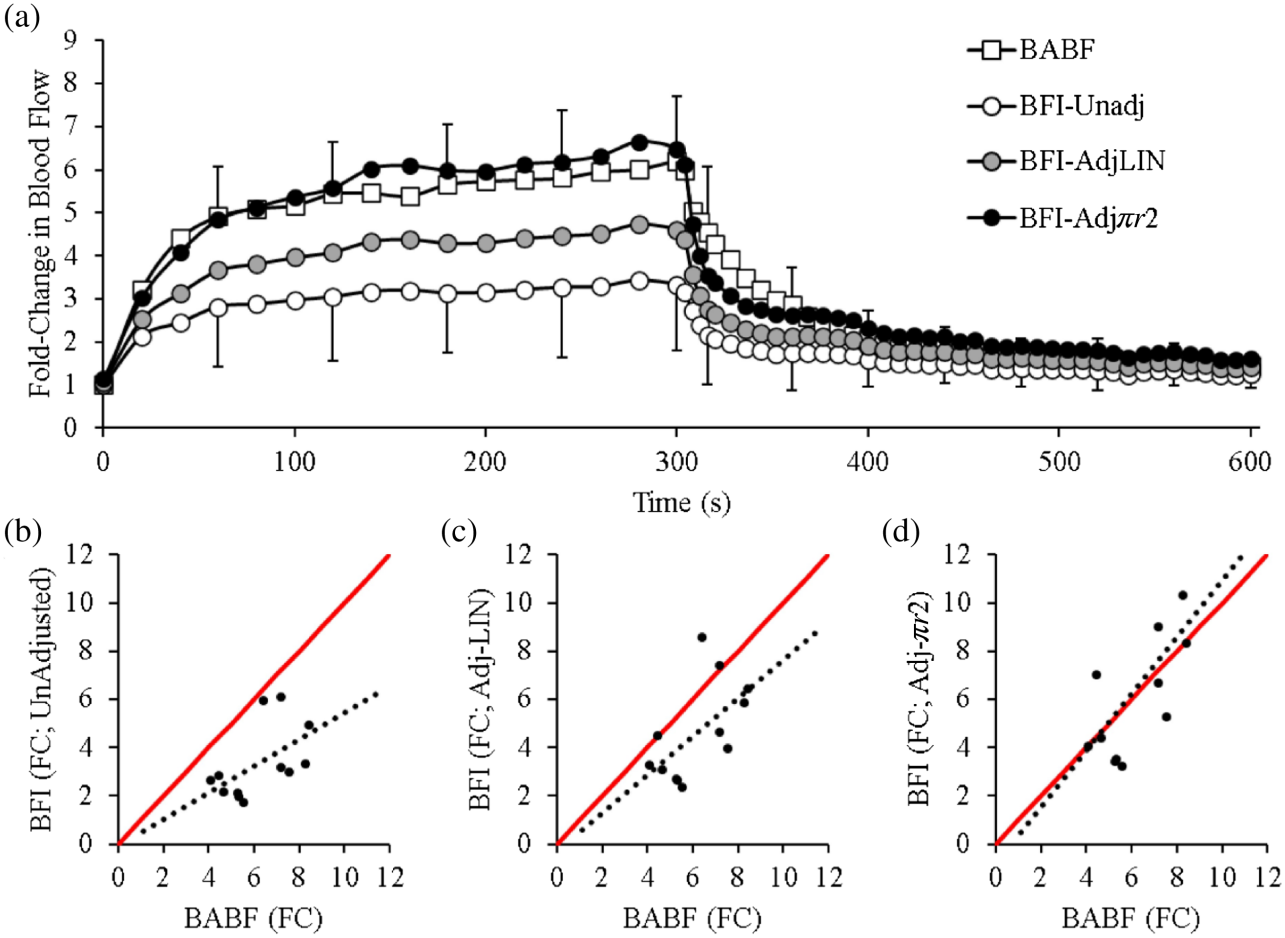
Adjusting DCS BFI for changes in microvascular area (${\mathrm{MV}}_{A}$). During handgrip exercise, the fold-change in BFI was either: (1) left unadjusted, (2) linearly adjusted for estimated changes in ${\mathrm{MV}}_{A}$ (${\mathrm{Adj}}_{\mathrm{LIN}}$), or (3) adjusted for changes in ${\mathrm{MV}}_{A}$ squared (${\mathrm{Adj}}_{\pi r2}$). Whereas the unadjusted (b) calculations underestimated the fold-change in brachial artery blood flow [(a), (b) p=0.002; BABF] at the end of handgripping, the ${\mathrm{Adj}}_{\mathrm{LIN}}$ [(c) p=0.073] and ${\mathrm{Adj}}_{\pi r2}$ [(d) p=0.743] calculations were not different from BABF. The line of identity was best, however, when using the ${\mathrm{Adj}}_{\pi r2}$ calculation. n=12 for all panels.

Accordingly, we investigated whether adjusting BFI for changes in microvascular flow area [Eqs. (2)–(4)] would reduce this discrepancy by leveraging an existing dataset that simultaneously measured changes in forearm BFI, NIRS-derived HbTot, and BABF during handgrip exercise.^8^ As shown in Fig. 2, the fold-change in BABF with exercise was markedly higher than the fold-change in the unadjusted BFI signal. However, using the increase in HbTot to adjust BFI for estimated changes in microvascular flow area (i.e., $\Delta {\mathrm{MV}}_{A}$) reduced this discrepancy, such that the $\mathrm{BFI}\text{-}{\mathrm{Adj}}_{\mathrm{LIN}}$ and $\mathrm{BFI}\text{-}{\mathrm{Adj}}_{\pi r2}$ were not different from BABF at the end of handgrip exercise (p=0.073 and p=0.743, respectfully). Moreover, the $\mathrm{BFI}\text{-}{\mathrm{Adj}}_{\pi r2}$ values were almost superimposed upon the BABF measures temporally and in magnitude. The line of identity between end-exercise BABF and $\mathrm{BFI}\text{-}{\mathrm{Adj}}_{\pi r2}$ was also close to 1.0, where as the unadjusted BFI and $\mathrm{BFI}\text{-}{\mathrm{Adj}}_{\mathrm{LIN}}$ calculations were much lower [Figs. 2(b)–2(d)].

**Fig. 3:**
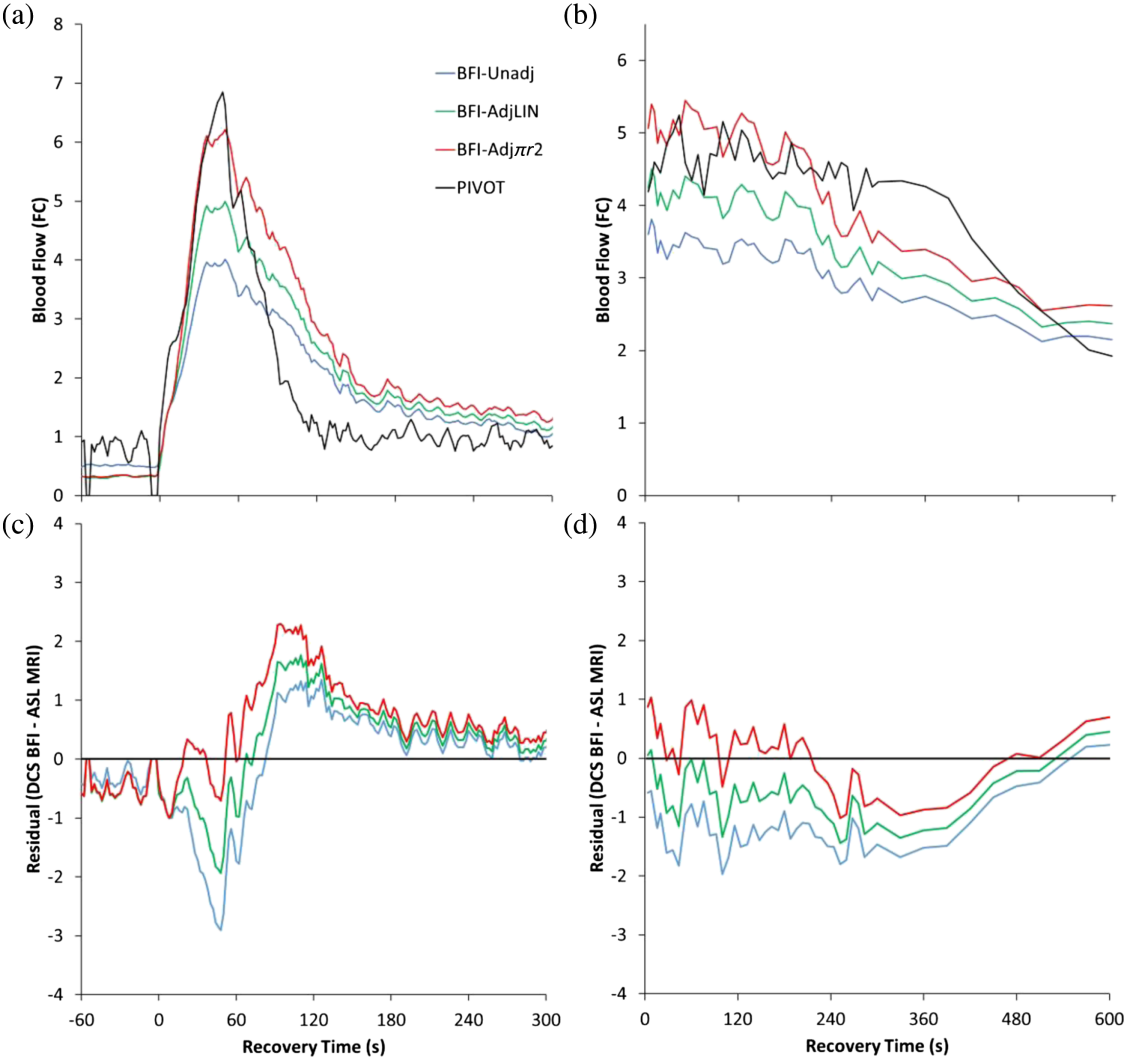
NIRS-DCS BFI versus ASL-MRI. We compared DCS BFI calculations of muscle perfusion against arterial spin-labeling MRI during (a) reactive hyperemia and (b) plantar flexion exercise. The unadjusted BFI calculation consistently underestimated ASL-measures of muscle perfusion, whereas the ${\mathrm{Adj}}_{\mathrm{LIN}}$ and $\mathrm{BFI}\text{-}{\mathrm{Adj}}_{\pi r2}$ calculations were much closer. (c), (d) Mean residuals (BFI – ASL) throughout the recovery periods.

As a final consideration, we compared DCS BFI calculations against ASL MRI (Fig. 3). Although changes in BFI were temporally aligned with changes in ASL during reactive hyperemia [Fig. 3(a)], the peak fold-change from the unadjusted BFI calculations were ∼½ the peak fold-change in ASL, which is consistent with data presented in Yu et al.^7^ In contrast, the peak fold-change from the $\mathrm{BFI}\text{-}{\mathrm{Adj}}_{\pi r2}$ calculation was very close to that of ASL. Similar observations were made following plantar flexion exercise, with the unadjusted BFI calculation underestimating ASL measures of muscle perfusion, but the $\mathrm{BFI}\text{-}{\mathrm{Adj}}_{\mathrm{LIN}}$ and $\mathrm{BFI}\text{-}{\mathrm{Adj}}_{\pi r2}$ calculations of muscle perfusion more closely matching those from ASL both temporally, and in magnitude [Fig. 3(b)].

Overall, the results presented here support *in silico* work by Boas et al.^5^ and *in situ* results from Sathialingam et al.,^6^ which demonstrated that changes in DCS BFI are susceptible to changes in ${\mathrm{MV}}_{A}$. Similarly, given that changes in ${\mathrm{MV}}_{A}$ will directly affect the shear-diffusion dynamics of RBCs, it is likely that changes in shear-diffusion also affect changes in DCS BFI within skeletal muscle. However, additional work is needed to further validate the BFI-adjustment calculations presented in this Letter [Eqs. (2)–(4)]. For example, as shown in Figs. 3(c) and 3(d), none of the DCS BFI calculation methods matched ASL-MRI throughout the recovery period. Although the $\mathrm{Adj}\text{-}\pi r^{2}$ calculation best matched ASL-MRI following plantar flexion exercise [Fig. 3(d)] and the peak of reactive hyperemia [Fig. 3(c)], it was the least-accurate method over the final ∼3  min of reactive hyperemia. Moreover, ASL-MRI measures of muscle perfusion recovered much faster than all 3 DCS BFI methods during reactive hyperemia.

The mechanism(s) underlying these discrepancies are not readily apparent but could be due to partial-volume effects. Notably, ASL-MRI measures of blood flow emanated exclusively from the medial gastrocnemius, whereas DCS BFI measures of “muscle” blood flow may have been affected by the overlying skin and adipose tissues that photons must first travel through before reaching the underlying muscle. Our recent work suggests that skin blood flow does not affect DCS BFI under thermoneutral conditions, but we cannot exclude the possibility that local heating may have stimulated increases in cutaneous perfusion that affected DCS BFI. Similarly, ASL-MRI measures of muscle perfusion were measured via the axial plane, whereas the DCS laser-detector orientation was longitudinal due to spatial/setup restrictions within the MRI-scanner. Future works will need to focus on better matching the tissue volumes measured by ASL-MRI and DCS BFI.

Our DCS signal intensity was above the estimated minimum threshold of ∼15,000 photon counts/second required for our MetaOx setup and sampling frequency.^15^ However, because reactive hyperemia and exercise increase total heme-content, and therefore near-infrared photon absorption ($\mu_{a}$), our results may have been affected by signal intensity. Additional work is needed to optimize NIRS-DCS devices and probes for use in skeletal muscle, such as utilizing longer wavelengths (e.g., 1064 nm) for DCS BFI,^16^ that permit long source-detector separations and maximize depth of penetration into skeletal muscle.

NIRS-based considerations may also have affected the results. Although hematocrit affects DCS BFI *in silico* and *in vitro*,^5^^,^^6^ to the best of our knowledge, no one has investigated how changes in hematocrit affect DCS BFI *in vivo*. Accordingly, future works should aim to directly manipulate hematocrit (e.g., isovolumic hemodilution^17^) and examine its impact on DCS BFI. Similarly, our BFI adjustment calculations depend on calculations of the heme-nadir, which could be affected by myoglobin, as this metabolite is indistinguishable from hemoglobin within the near-infrared spectrum.^18^ Accordingly, more work is needed to assess how myoglobin affects calculation of the heme-nadir, as well as work that establishes standard methods for measuring $\Delta {\mathrm{MV}}_{A}$ across individuals and populations.

## 4 Conclusions

The results presented herein provide novel empirical evidence that relative changes in DCS BFI do not translate directly to relative changes in muscle perfusion, but that adjusting relative changes in BFI for estimated changes in microvessel flow area may help overcome this issue. However, additional work is needed to further validate the BFI-Adj calculations presented herein, which will likely require substantial interdisciplinary collaboration between physicists, engineers, and physiologists alike to optimize NIRS-DCS devices and probes for use in skeletal muscle.

## Data Availability

Data are available from the authors upon reasonable request.
